# Professional Notes

**Published:** 1887-12-24

**Authors:** Geo. W. Potter


### Professional Notes.

By Geo. W. Potter, M.D.

The Bacillus Tuberculosis does not diminish either in interest or importance as knowledge of the subject increases. The ablest observers and clinicians are quite at one as to its significance in tubercular disease. Professor Dujardin-Beaumetz discusses, in the November number of the *Therapeutic Gazette*, certain *new antiseptic methods of treating pulmonary diseases*, all based on the assumption that the efficient agent in the production of certain of those diseases is the *bacillus tuberculosis*. A Frenchman is nothing if not definite, and Dr. Dujardin-Beaumetz is every inch a Frenchman. Believing, as he does, that the cause of pulmonary phthisis is the bacillus, he sets to work logically to kill the bacillus, and so to put an end to the phthisis, on the excellent principle that if the cause be removed the effect will cease. There is a decided advantage in this definite habit of mind, inasmuch as it prompts to the performance of test experiments, and so gives positive results, which either justify or discredit the assumptions on which the experiments are founded. In physical science the only methods of verifiable progress are those of observation and experiment. Before the adoption of those methods medicine for centuries moved only in a circle. Positive advance along the whole line was impossible. In order that experiments similar to those of Dr. Dujardin-Beaumetz may be carried out on rational lines it is necessary to premise certain conditions. First, a "perfect knowledge of the microbe itself, and the conditions in which it developes, reproduces itself, and dies, is indispensable. We must know, too, its degree of resistance, and especially its degree of vitality as contrasted with that of the organ where it vegetates; and this last condition is practically the most important of all. In fact, the lung is an organ pre-eminently essential to life; and if, in attempting to effect destruction of the germ of the disease, you injure the organ which is its habitat, you will have left the subject in a worse state than before. Unfortunately the tubercle bacillus is a micro-organism characterised by great resistance, and difficult to overcome;" able to "tolerate very energetic treatment." A number of the antiseptics used for inhalations "are irritant substances, whose use cannot be prolonged with impunity, and which soon determine in the larynx, trachea, bronchi, and even in the lungs, symptoms of irritation and inflammation which cannot but be prejudicial to the patient. Moreover, the lungs are an effective channel for absorption into the blood, and as many of the best antiseptics are toxic, and the patient in carrying out the treatment would necessarily inhale considerable quantities, there would be more or less danger of systemic poisoning. Lastly, . . . "recent experiments have proved that in inhalation atomised saline or other substances penetrate with difficulty to the last bronchial ramifications, and that the *greater part of these sprays are retained in the upper air passages*. The physiological experiments on animals undertaken by Professor Jacobelli, of Naples, . . . before the Commission appointed by the Academy, have shown how questionable a matter is this penetration of vaporised solutions to the ultimate bronchi." . . . Marey and Brouardel have expressed similar doubts on the subject.

The germicide treatment of pulmonary phthisis by inhalations is beset with difficulties. To avoid these, Dr. Dujardin-Beaumetz seeks to avail himself "of a property possessed by the pulmonary textures of *elimination by exhalation through the air cells* of substances introduced into the blood. Medicament must be introduced hypodermically, or by the stomach or rectum." The stomach in phthisical patients is seldom available, for many reasons, and therefore "we are obliged to choose the hypodermic method, or avail ourselves of the property of absorption possessed by the rectum." Substances thus introduced by hypodermic injection or by the rectum, after entering the circulation, are partly eliminated by the lungs, and in the process of elimination "come in contact with the diseased parts, and thus enter into conflict with the morbid germ." It is clear that by either of these methods not only are irritation and inflammation of the air passages avoided, but also that the germicide will infallibly reach the actual habitat of the organism to be destroyed. The substance employed will thus have at least a chance of effecting the object intended, and, provided it have no general toxic properties, it is difficult to see what harm it can do.

Phenic acid hypodermically injected, is a germicide on which Dr. Dujardin-Beaumetz places much reliance in tubercular phthisis. It is highly volatile, and is freely eliminated by the lungs. Its antiseptic properties are very marked. As well as by Dujardin-Beaumetz himself, experiments have been made with it by Dr. Filleau, Dr. Leon Petit, and Dr. Sapelier. "The phenic acid injections may be made directly under the skin, or the needle may be plunged deeply into the soft parts, and even to the seat of the lesion when the injection is made in the region of the thorax. An ordinary hypodermic syringe is inadequate for these deep injections of phenic acid, and it is necessary to make use of one of a larger kind. A two per. cent. solution dissolved in glycerine is found suitable. An alcoholic solution is too irritant. The place for the injection is the anterior part of the chest under the clavicle. The punctures should be made more or less often . . . from two each week to two each day. Under no circumstances should they be too often repeated, for in exceeding a certain dose "there is danger of seeing all the symptoms of carbolic poisoning—coldness, cyanosis, collapse, vomiting, etc. Moreover, some persons are particularly intolerant of this medicinal agent, and cannot with safety be subjected to this treatment."

Results are what must always be the ultimate criteria when new methods of treatment are brought up for judgment. "The injections of phenic acid for phthisis have," says Dr. Dujardin-Beaumetz, "given quite favourable results in a certain number of cases. Almost always the appetite is improved; patients that previously were bedridden are able to get up and go out and take the air—a great advantage in consumption. The cough and expectoration have diminished, and . . . we have often seen night sweats disappear." That seems to be the most the French Professor is able to affirm; and it is of no small importance. But unless larger and more permanent results should be obtained by other experimenters it cannot be said that a cure for phthisis has been discovered. All the ameliorations of the patient's condition claimed for the phenic acid injections have been claimed for a dozen other substances, used in a dozen different ways, and with quite as much justice. Inhalations, injections, medicaments administered by the mouth and by the rectum, and indeed every method of treatment practised in phthisis, affirms its measure of success. But as yet no method has been employed in practice which can command the just confidence of practitioners in every country. We are groping after the truth with all the ardour and energy of which we are capable. But, in spite of all past efforts, there still remains to be won the signal honour of discovering a real and veritable cure for what is perhaps the direst physical scourge of the human race. There is no reason to despair of success, even though so many efforts have been comparatively fruitless. There are among the medical men of the present time some who to the determination of a Wellington unite the clear intellect and patience of a Newton; and it will be strange indeed if they and their successors do not carry forward the torch of science until they finally reach the daylight of complete knowledge and successful practice.

